# Euthyroid Sick Syndrome in Patients With COVID-19

**DOI:** 10.3389/fendo.2020.566439

**Published:** 2020-10-07

**Authors:** Runmei Zou, Chenfang Wu, Siye Zhang, Guyi Wang, Quan Zhang, Bo Yu, Ying Wu, Haiyun Dong, Guobao Wu, Shangjie Wu, Yanjun Zhong

**Affiliations:** ^1^Children’s Medical Center, The Second Xiangya Hospital, Central South University, Changsha, China; ^2^Critical Care Medicine, The Second Xiangya Hospital, Central South University, Changsha, China; ^3^Critical Care Medicine, The First Hospital of Changsha, Changsha, China; ^4^Department of Respiratory, The Second Xiangya Hospital, Central South University, Changsha, China

**Keywords:** Coronavirus Disease 2019 (COVID-19), euthyroid sick syndrome, thyroid function, disease severity, C-reactive protein

## Abstract

**Background:**

Coronavirus disease 2019 (COVID-19) has been shown to affect almost every organ throughout the body. However, it is not clear whether the thyroid gland is impaired in COVID-19 patients. Euthyroid sick syndrome (ESS) is usually associated with the disease severity and deterioration prognosis in critical illness. In this study, the thyroid function of COVID-19 patients was assessed and factors associated with outcomes were analyzed to determine the potential predictive value of ESS.

**Methods:**

Clinical and laboratory data of COVID-19 patients with or without ESS in Changsha, China, were collected and analyzed on admission. Kaplan-Meier curve and cox regression model were utilized to determine the correlation between ESS and the endpoints. Subsequently, a receiver operating characteristic (ROC) curve was plotted to evaluate the predictive performances of FT3 and C-reactive protein (CRP) in the disease severity.

**Results:**

Forty-one (27.52%) cases of COVID-19 patients diagnosed with ESS. ESS patients had higher proportions of fever, shortness of breath, hypertension, diabetes, and severe events than those of non-ESS patients. The levels of erythrocyte sedimentation rate and C-reactive protein, and the positive rate of procalcitonin were significantly higher, whereas the lymphocyte count was apparently lower in ESS patients than in non-ESS patients. The regression analysis showed that ESS was significantly associated with the disease severity of COVID-19 (HR = 2.515, 95% CI: 1.050–6.026, *P* = 0.039). The areas under the curve (AUCs) for predicting the severe disease were [0.809 (95% CI 0.727–0.892), *P* < 0.001] and [0.792 (95% CI 0.689–0.895), *P* < 0.001] for FT3 and CRP, respectively.

**Conclusion:**

ESS was significantly associated with the disease severity and inflammatory parameters in COVID-19 patients.

## Introduction

Coronavirus Disease 2019 (COVID-19) is an infectious disease which causes severe respiratory illness. It was first reported in Wuhan, China, in December 2019 (1–3). It has spread widely around the world and been declared a pandemic by the World Health Organization (WHO) on March 11, 2020. As of August 28, over 24,000,000 cases and 800,000 deaths have been identified worldwide (4). The etiological agent of COVID-19 has been confirmed as a novel coronavirus, initially named 2019-nCov but now known as severe acute respiratory syndrome coronavirus 2 (SARS-CoV-2) (5).

COVID-19 can impair almost any structure in the human body from the brain to the toes, including lungs, liver, heart, kidney, brain, gut, etc. (6). The thyroid gland is a neuroendocrine organ which plays an important role in regulating immunity and metabolism. Whether the thyroid gland is affected by SARS-CoV-2 remains unclear. An identity match of more than 85% has been identified between SARS-CoV-2 and a bat SARS-like CoV genome published previously (7). A study composed of 48 SARS patients found that 93.7% patients had a low serum level of triiodothyronine (T3) (8). During acute illness, changes in the serum levels of thyroid hormones have been depicted which represent a condition known as euthyroid sick syndrome (ESS). ESS is characterized by a decreased level of serum T3 and/or thyroxine (T4) without an increased secretion of thyroid-stimulating hormone (TSH) (9). ESS is the physiologic adaptation and pathologic response to acute disease, occurring in the fasting state in healthy individuals, as well as in the context of infection, trauma, myocardial infarction, and malignancy (10). Previous studies suggested that thyroid hormone levels, especially low serum levels of free T3 (FT3), are usually associated with the disease severity and deterioration prognosis in critical illness (11, 12).

In this study, we assessed the thyroid function in patients with COVID-19 and analyzed the related factors to determine the potential value of ESS in predicting the disease severity. We found that COVID-19 patients with ESS had a higher risk of severe events than non-ESS patients. Besides, ESS was an independent risk factor for the severe event in COVID-19 patients. ESS also displayed a potent correlation with the inflammatory parameters.

## Methods

### Study Design and Participants

This was a retrospective cohort study. The study was approved by the institutional ethics board of the second Xiangya Hospital of Central South University (No. 2020001). Laboratory-confirmed COVID-19 patients (aged 19–84 years old) admitted to Public Health Treatment Center of Changsha, China from January 17 to March 14, 2020, were enrolled. Patients with underlying primary thyroid disease, history of chemotherapy or radiotherapy in the last 6 months, suspicion of underlying hypothalamic or pituitary disease were excluded. ESS was described as serum FT3 <2.3 pg/ml with low or normal levels of TSH. Patients were divided into two groups according to serum FT3 values: ESS group and non-ESS group.

### Data Collection

Two members of our team carefully gathered and individually reviewed the medical records of patients. Detailed information on demographic data, clinical symptoms, underlying comorbidities, medical history, laboratory parameters, and chest computed tomographic (CT) scans were checked and extracted. Blood samples were drawn from the patients on their first day of the hospitalization.

### Endpoints

The primary endpoint of our study was the severe event. The second endpoints were the rates of noninvasive and invasive ventilation, mortality, virus shedding time, and length of hospital stay. We used one of the following criteria to determine the severe event of COVID-19: 1) respiratory rate ≥30/min; 2) oxygen saturation ≤93%; 3) PaO2/FiO2 ≤300 mmHg; 4) progression of lung lesions exceeding 50% within 24–48 h; 5) mechanical ventilation was implemented; 6) shock; 7) intensive care unit admission (13). The virus shedding time was defined as the time from illness onset (the day of diagnosis for asymptomatic patients) to the first negative samples without any positive sample thereafter.

### Statistical Analysis

Continuous variables in our study were expressed as the median with inter quartile range (IQR) for data not following normal distribution and analyzed using the Mann-Whitney U test. Categorical data were described using frequencies and percentages. The χ2 test or Fisher’s exact test was used to compare the differences of the categorical variables. The Kaplan-Meier (KM) curve and cox proportional hazard regression models were applied to determine the association between ESS and the endpoints, with the hazards ratio (HR) and 95% confidence interval (95% CI) being reported. Finally, a receiver operating characteristic (ROC) curve was established to evaluate the predictive performances of FT3 and C-reactive protein (CRP) in the disease severity of COVID-19.

## Results

A total of 149 COVID-19 patients were enrolled in this study, of whom 41 (27.52%) cases were diagnosed with ESS. Out of these, 14 (34.15%) were males. The median age was 58 (IQR: 50–66) years old. The most common symptoms in the ESS group were fever [39 (95.12%)], fatigue [18 (43.90%)], cough [36 (87.80%)], shortness of breath [25 (60.98%)], expectoration [20 (48.78%)], and anorexia [21 (51.22%)]. Hypertension [10 (24.39%)] and diabetes [7 (17.07%)] were the most common comorbidities (**Table 1**).

**Table 1:** Baseline characteristics of patients with ESS and non-ESS.

	ESS group (n = 41)	Non-ESS group (n = 108)	All patients (n = 149)	*P* value
Gender (male/female)	14/27	57/51	71/78	0.042*
Age, y, M (IQR)	58 (50, 66)	41 (31, 56.5)	47 (36, 61.5)	<0.001*
**Symptoms**				
Fever (n, %)	39 (95.12)	73 (67.59)	112 (75.17)	0.001*
Fatigue (n, %)	18 (43.90)	43 (39.81)	61 (40.94)	0.650
Cough (n, %)	36 (87.80)	88 (81.48)	124 (83.22)	0.356
Shortness of breath (n, %)	25 (60.98)	26 (24.07)	51 (34.23)	<0.001*
Expectoration (n, %)	20 (48.78)	49 (45.37)	69 (46.31)	0.709
Hemoptysis (n, %)	3 (7.32)	2 (1.85)	5 (3.36)	0.128
Pharyngalgia (n, %)	6 (14.63)	21 (19.44)	27 (18.12)	0.496
Vomiting (n, %)	4 (9.76)	12 (11.11)	16 (10.74)	1.000
Diarrhea (n, %)	8 (19.51)	24 (22.22)	32 (21.48)	0.719
Abdominal pain (n, %)	3 (7.32)	2 (1.85)	5 (3.36)	0.128
Nausea (n, %)	5 (12.20)	12 (11.11)	17 (11.41)	1.000
Anorexia (n, %)	21 (51.22)	47 (43.52)	68 (45.64)	0.399
Myalgia (n, %)	3 (7.32)	11 (10.19)	14 (9.40)	0.825
Chill (n, %)	8 (19.51)	12 (11.11)	20 (13.42)	0.179
Dizziness (n, %)	7 (17.07)	12 (11.11)	19 (12.75)	0.330
Headache (n, %)	7 (17.07)	16 (14.81)	23 (15.44)	0.733
**Comorbidities**				
Hypertension (n, %)	10 (24.39)	7 (6.48)	17 (11.41)	0.005*
Cardiovascular (n, %)	3 (7.32)	4 (3.70)	7 (4.70)	0.619
Diabetes (n, %)	7 (17.07)	3 (2.78)	10 (6.71)	0.006*
Chronic liver disease (n, %)	1 (2.44)	6 (5.56)	7 (4.70)	0.712

*means a significant difference; ESS, euthyroid sick syndrome; M, median; IQR, inter quartile range; y, years.

Compared with non-ESS patients, patients with ESS were older [58 years (IQR: 50–66) *vs* 41 years (IQR: 31–56.5), *P* < 0.001) and exhibited a female dominance (65.85 *vs* 47.22%, *P* = 0.042). Furthermore, patients with ESS tended to have a higher prevalence of fever and shortness of breath compared to non-ESS patients (95.12 *vs* 67.59%, *P* = 0.001; 60.98 *vs* 24.07%, *P* < 0.001, respectively). The occurrences of hypertension and diabetes were higher in ESS patients than those in non-ESS patients (24.39 *vs* 6.48%, *P* = 0.005; 17.07 *vs* 2.78%, *P* = 0.006, respectively) (**Table 1**).

Patients with ESS had significantly lower levels of T4 (median 106.30 *vs* 121.98 nmol/L, *P* < 0.001), and free T4 (FT4) (median 14.47 *vs* 16.08 pmol/L, *P* = 0.026) than non-ESS patients, but no significant difference was detected with respect to TSH. The levels of erythrocyte sedimentation rate (median 66.5 *vs* 35 mm/h, *P* < 0.001) and CRP (median 38.99 *vs* 11.26 mg/L, *P* < 0.001) were significantly higher, whereas the lymphocyte count (0.91 *vs* 1.27 ×10^9^/L, *P* <0.001) was obviously lower in ESS patients than those of non-ESS patients. Additionally, patients with ESS had a higher positive rate of procalcitonin compared with non-ESS patients (53.66 *vs* 29.63%, P = 0.006) (**Table 2**). COVID-19 patients with ESS were linked with stronger inflammatory responses, characterized by higher levels of inflammatory markers.

**Table 2:** Laboratory findings of patients with ESS and non-ESS.

	ESS group (n = 41)	Non-ESS group (n = 108)	All patients (n = 149)	*P* value
WBC, ×10^9^/L, M (IQR)	4.62 (3.38, 6.15)	4.67 (3.68. 5.98)	4.63 (3.60, 6.03)	0.708
Lymphocyte count, ×10^9^/L, M (IQR)	0.91 (0.59, 1.13)	1.27 (0.91, 1.70)	1.13 (0.83, 1.52)	<0.001*
T3, nmol/L, M (IQR)	1.01 (0.81, 1.10)	1.47 (1.35, 1.68)	1.39 (1.11, 1.59)	<0.001*
T4, nmol/L, M (IQR)	106.30 (89.38, 119.09)	121.98 (107.78, 136.13)	118.80 (100.67, 131.15)	<0.001*
FT4, pmol/L, M (IQR)	14.47 (12.72, 17.18)	16.08 (13.43, 18.91)	15.80 (13.35, 18.11)	0.026*
TSH, uIU/ml, M (IQR)	1.36 (1.01, 2.28)	1.74 (1.13, 2.71)	1.63 (1.07, 2.56)	0.065
ESR, mm/h, M (IQR)	66.5 (43, 82.5)	35 (15, 60)	46 (21.5, 68.5)	<0.001*
CRP, mg/L, M (IQR)	38.99 (19.05, 60.96)	11.26 (2.92, 22.88)	16.07 (4.14, 34.07)	<0.001*
PCT ≥0.05 ng/ml, (n, %)	22 (53.66)	32 (29.63)	54 (36.24)	0.006*
ALT, U/L, M (IQR)	18.95 (13.37, 28.75)	19.72 (14.11, 26.41)	19.08 (13.75, 26.44)	0.973
AST, U/L, M (IQR)	25.05 (21.31, 34.82)	23.84 (18.85, 29.19)	23.97 (19.38, 30.47)	0.098
Tbil, μmol/L, M (IQR)	11.14 (7.73, 14.59)	10.29 (8.20, 14.82)	10.60 (8.08, 14.68)	0.784
SCr, mmol/L, M (IQR)	54.91 (37.67, 65.55)	50.51 (41.05, 65.66)	51.11 (40.74, 65.43)	0.883
CK, U/L, M (IQR)	75.90 (50.10, 139.10)	68.25 (44.38, 105.63)	68.70 (45.40, 111.40)	0.321
CK-MB, M (IQR)	11.00 (6.35, 13.95)	9.50 (6.10, 12.30)	10.00 (6.10, 12.99)	0.320
Chest CT positive rate (n, %)	41 (100.00)	101 (93.52)	142 (95.30)	0.216

*means a significant difference; ESS, euthyroid sick syndrome; M, median; IQR, inter quartile range; WBC, white blood cell count; T3, triiodothyronine; T4, thyroxine; FT4, free thyroxine; TSH, stimulating hormone; ESR, erythrocyte sedimentation rate; CRP, C-reactive protein; PCT, procalcitonin; ALT, alanine aminotransferase; AST, aspartate aminotransferase; TBil, total bilirubin; SCr, serum creatinine; CK, creatine kinase; CK-MB, creatine kinase isoenzyme.

**Figure 1:**
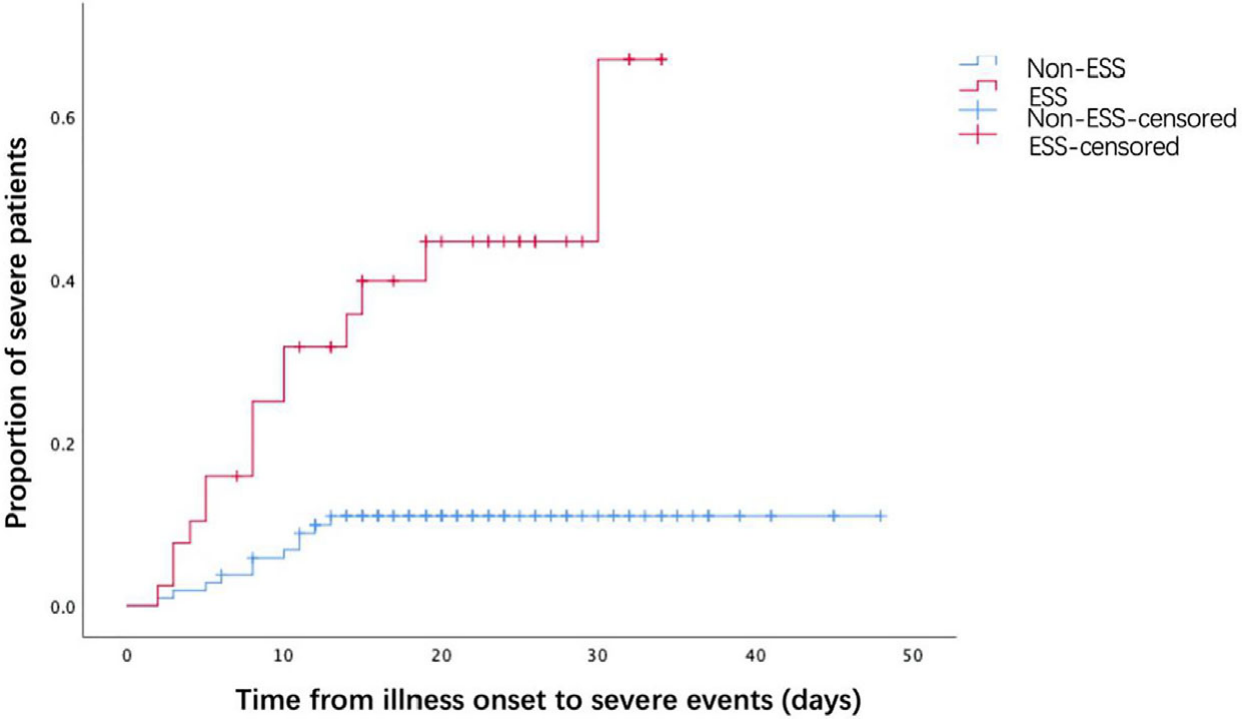
The time-dependent risk of reaching to the severe events.

The associations between ESS and the outcomes of COVID-19 patients were presented in **Table 3**. COVID-19 patients with ESS had a significantly higher prevalence of severe events (36.59 *vs* 10.19%, *P* < 0.001). Nonetheless, no significant effects of ESS were found on the rates of noninvasive (2.44 *vs* 1.85%, *P* = 1.000) and invasive ventilation (2.44 *vs* 0.93%, *P* = 0.476), mortality (0.00 *vs* 0.93%, *P* = 1.000), virus shedding time [19 days (IQR: 14–23.5) *vs* 17 days (IQR: 13–24), *P* = 0.670], and length of hospital stay [17 days (IQR:12–25) *vs* 15 days (IQR: 11–23), *P* = 0.345]. As observed from the KM curve, patients with ESS had a significantly higher risk of severe events compared with those without ESS (log-rank *P* < 0.001, **Figure 1**). The results of cox regression analysis showed that both ESS (HR = 2.515, 95% CI: 1.050–6.026, *P* = 0.039), and CRP (HR = 1.031, 95% CI: 1.015–1.046, *P* < 0.001) were independent risk factors for the disease severity (**Table 4**).

**Table 3:** Outcomes of patients with ESS and non-ESS.

	ESS group (n = 41)	Non-ESS group (n = 108)	All patients(n = 149)	*P* value
Severe event (n, %)	15 (36.59)	11 (10.19)	26 (17.45)	<0.001*
Noninvasive ventilator (n, %)	1 (2.44)	2 (1.85)	3 (2.01)	1.000
Invasive ventilator (n, %)	1 (2.44)	1 (0.93)	2 (1.34)	0.476
Mortality (n, %)	0 (0.00)	1 (0.93)	1 (0.67)	1.000
Virus shedding time (days, IQR)	19 (14, 23.5)	17 (13, 24)	17.50 (13, 24)	0.670
Length of hospital stay (days, IQR)	17 (12, 25)	15 (11, 23)	15.5 (11, 24.75)	0.345

*means a significant difference; ESS, euthyroid sick syndrome; IQR, inter quartile range.

**Table 4:** Association of ESS and disease severity in the cox regression model.

	Beta	SE	Wald	*P* value	Hazard ratio	95.0% CI	
						Lower	Upper
ESS	0.922	0.446	4.280	0.039*	2.515	1.050	6.026
Gender	0.313	0.456	0.472	0.492	1.368	0.559	3.345
Age	0.006	0.017	0.147	0.701	1.006	0.974	1.040
Hypertension	0.417	0.549	0.576	0.448	1.517	0.517	4.447
Cardiovascular disease	-0.777	0.579	1.800	0.180	0.460	0.148	1.431
CRP	0.030	0.008	15.246	<0.001	1.031	1.015	1.046

*means a significant difference; ESS, euthyroid sick syndrome; CRP, C-reactive protein.

**Figure 2:**
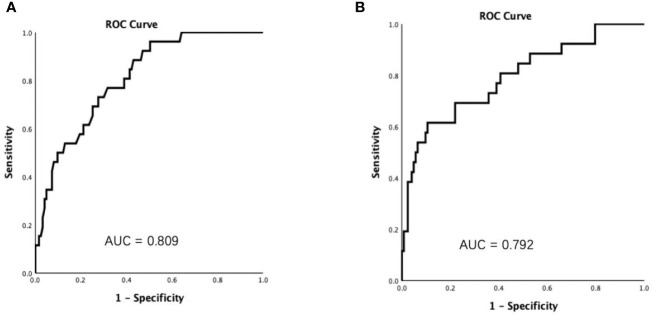
Receiver operating characteristic (ROC) curves showed that the areas under the curve (AUCs) were 0.809 and 0.792 for FT3 **(A)** and C reactive protein **(B)**, respectively.

Considering that CRP was one of the most reported risk factors of the disease severity of COVID-19 patients (14–17), ROC curve was conducted to determine the predictive performances of FT3 and CRP in the disease severity. The areas under the curve (AUCs) for FT3 and CRP predicting the severe disease were [0.809 (95% CI 0.727–0.892), *P* < 0.001] and [0.792 (95% CI 0.689–0.895), *P* < 0.001] (**Figure 2**), respectively.

## Discussion

In the present study, we identified 41 (27.52%) COVID-19 patients with ESS. We compared the clinical characteristics between ESS and non-ESS patients and analyzed factors related to the disease severity of patients with COVID-19. The results demonstrated that ESS patients had a higher prevalence of severe events than non-ESS patients, which was consistent with the severity of symptoms and elevated inflammatory markers. The cox regression model suggested that ESS is an independent risk factor for the severity of COVID-19.

Previous studies demonstrated that a decreased level of FT3 was a prognostic indicator of critical disease, which was associated with all-cause mortality, especially in the intensive care unit (18). A study consisting of 503 patients diagnosed with community-acquired pneumonia reported that ESS is an independent risk factor for the 30-day mortality (19). The pathophysiological process of ESS involved in the induction of type III deiodinase and decrease of type I deiodinase, which resulted in the increased conversion of T4 to rT3 instead of T3 (9). Furthermore, the HPT axis was suppressed under pathological conditions, contributing to the decreased secretion of TSH (20). This pathophysiological mechanism also involved the binding of thyroid hormone to plasma protein, transport of thyroid hormone in peripheral tissues, and the hormone receptor activity (10).

In COVID-19 patients, ESS may be directly caused by infection of thyroid cells with SARS-CoV-2. Despite the fact that no article on the involvement of the thyroid gland in COVID-19 patients has been published, varying degrees of damage to the thyroid gland were confirmed in SARS patients back in 2007 (21). Meanwhile, the patients with COVID-19 had lower serum TSH and TT3 levels than healthy controls and non-COVID-19 pneumonia patients (19). Angiotensin-converting enzyme (ACE) 2, an important receptor in the pathogenesis of COVID-19, was demonstrated to be expressed in the thyroid tissues (22). Hence, the thyroid gland may be one of the targets damaged by SARS-CoV-2. Withal, the mechanism for this hypothesis remains unknown. Further investigation is necessary to determine whether ACE2 has an impact on the thyroid function in patients with COVID-19. Second, cytokine storms were very common in COVID-19 patients, especially in severe cases, characterized by the uncontrolled and excessive release of inflammatory mediators resulting in overwhelming systemic inflammation and even multiple organ dysfunction (23). In our research, the inflammatory responses seemed to be stronger in patients with ESS, with higher levels of CRP and erythrocyte sedimentation rate as well as a higher positive rate of procalcitonin. It is well known that cytokines are the key molecules involved in coordinating the hormone, immune, and inflammatory responses to stressful stimuli (24). In the previous study, patients admitted to the intensive care unit elicited lower serum concentrations of T4, FT4, T3, FT3, and TSH, while the serum levels of inflammatory cytokines including IL-1β and TNF-α were markedly elevated (25). The increase of inflammatory cytokines can result in suppression of central TSH and 5’-deiodinases activity. Third, our results indicated that ESS patients were more likely to have the symptom of fever compared with non-ESS patients. The invasion of SARS-CoV-2 causing hyperthermia can lead to down-regulation of 5’-deiodinases activity, resulting in decreases in T3 levels. Meanwhile, the disease related negative nitrogen balance and body consumption, can also lead to a decline in the serum levels of thyroid hormone transport proteins, inhibiting T4 transport in T3-producing tissues. Fourth, the effects of drugs on the thyroid function should not be ignored. Glucocorticoids and dopamine can inhibit the secretion of TSH by the pituitary and the intake of T4 by peripheral tissues. Likewise, amiodarone and beta-adrenergic blocking agents can suppress deiodinase activity and consequently T3 production, contributing to a decrease in serum T3 levels. Also, non-steroidal anti-inflammatory drugs (NSAIDs) are capable of transiently increasing free thyroid hormone levels by preventing their binding to plasma transport proteins (26). Given that the thyroid functions of our patients were assessed on admission, further studies including the effects of drugs on the thyroid function are imperative to demonstrate the impact of COVID-19 pharmacotherapy on the development of ESS.

Our work above had some limitations. First, this study was a retrospective study, and the thyroid function was accessed only at admission. Second, the sample size was too small and only a few patients required the mechanical ventilation. Based on this fact, the assessment of the correlation between ESS and the ventilation requirement performed in this study may be biased. Third, the levels of glucocorticoid, rT3 and the pituitary function were unknown, which are needed to exclude the effects of other factors on the functions of various endocrine glands.

In summary, more than 25% COVID-19 patients were diagnosed with ESS in our research. ESS is an independent risk factor for the disease severity of COVID-19. COVID-19 patients with ESS had stronger inflammatory responses, with higher levels of CRP and erythrocyte sedimentation rate as well as a higher positive rate of procalcitonin. Further investigation with a large sample size and an appropriate study design are needed to demonstrate the impact of ESS in COVID-19 patients.

## Data Availability Statement

The datasets presented in this article are not readily available because: The data used in this paper are from public health treatment center, which can only be obtained with the approval of relevant institutions. Requests to access the datasets should be directed to YZ, zhongyanjun@csu.edu.cn.

## Ethics Statement

The studies involving human participants were reviewed and approved by: The study was approved by the institutional ethics board of the Second Xiangya Hospital of Central South University (No. 2020001). Written informed consent for participation was not required for this study in accordance with the national legislation and the institutional requirements. Written informed consent was not obtained from the individual(s) for the publication of any potentially identifiable images or data included in this article.

## Author Contributions

RZ and CW were involved in study design, interpreting data, statistical analysis, creating tables and figures, and writing of the manuscript. SZ, GWa, QZ, BY, YW, and HD were involved in collecting data. YZ, SW, and GWu were involved in interpreting data, statistical analysis, and designed the research, supervised the work. All authors contributed to the article and approved the submitted version.

## Funding

This study was supported by Emergency project for COVID-19 prevention and control of Central South University, China (No 160260005).

## Conflict of Interest

The authors declare that the research was conducted in the absence of any commercial or financial relationships that could be construed as a potential conflict of interest.
